# Identifying Methylation Patterns in Dental Pulp Aging: Application to Age-at-Death Estimation in Forensic Anthropology

**DOI:** 10.3390/ijms22073717

**Published:** 2021-04-02

**Authors:** Sara C. Zapico, Quentin Gauthier, Aleksandra Antevska, Bruce R. McCord

**Affiliations:** 1 Department of Chemistry and Biochemistry and International Forensic Research Institute, Florida International University, Miami, FL 33199, USA; qgaut001@fiu.edu (Q.G.); mccordb@fiu.edu (B.R.M.); 2 Anthropology Department, National Museum of Natural History, Smithsonian Institution, Washington, DC 20560, USA; 3 Department of Chemistry, The University of Tennessee, Knoxville, Knoxville, TN 37996, USA; aantevsk@vols.utk.edu

**Keywords:** age-at-death estimation, adults, teeth, pulp, DNA methylation, ELOVL2, KLF14, NPTX2, SCGN, FHL2

## Abstract

Age-at-death estimation constitutes one of the key parameters for identification of human remains in forensic investigations. However, for applications in forensic anthropology, many current methods are not sufficiently accurate for adult individuals, leading to chronological age estimates erring by ±10 years. Based on recent trends in aging studies, DNA methylation has great potential as a solution to this problem. However, there are only a few studies that have been published utilizing DNA methylation to determine age from human remains. The aim of the present study was to expand the range of this work by analyzing DNA methylation in dental pulp from adult individuals. Healthy erupted third molars were extracted from individuals aged 22–70. DNA from pulp was isolated and bisulfite converted. Pyrosequencing was the chosen technique to assess DNA methylation. As noted in previous studies, we found that ELOVL2 and FHL2 CpGs played a role in age estimation. In addition, three new markers were evaluated—NPTX2, KLF14, and SCGN. A set of CpGs from these five loci was used in four different multivariate regression models, providing a Mean Absolute Error (MAE) between predicted and chronological age of 1.5–2.13 years. The findings from this research can improve age estimation, increasing the accuracy of identification in forensic anthropology.

## 1. Introduction

In forensic cases involving skeletal remains, the creation of a biological profile is essential to search missing persons reports, helping to determine potential matches and to assist in the identification of the victim [1]. Biological profiles commonly include sex, age, ancestry, and height. Thus, age estimation is one of the key components that is used for the identification of the remains. However, while age can be determined very accurately in childhood because it is based on the forensic anthropological assessment of growth and development [2,3,4,5,6,7], this estimation is less accurate in adult individuals. In adults, age assessment is based on degenerative changes in bones and teeth. This estimation requires a combination of different methods, such as the application of Lamendin’s formula in uniradicular teeth (canines and incisors) [8]; pubic symphysis assessment [9]; auricular surface evaluation [10]; fourth rib [11]. Using these methodologies can result in a general estimate of age with an accuracy of ±10 years. However, these results can be affected by endogenous and exogenous factors, pathological conditions, and in mass disasters, fragmentary remains [1].

The application of biochemical techniques, based on the natural process of aging, may provide a potential solution to this problem [12]. Until recently, the racemization of aspartic acid in dentin seemed to be the most accurate technique to determine the age of adult individuals [13,14,15,16]. However, epigenetics, the study of heritable changes in gene expression [17,18], has been shown to be a valuable and more accurate tool for age estimation [19]. Epigenetic DNA methylation, which involves the addition of a methyl group to a cytosine nucleotide in cytosine/guanine regions, known as CpG islands, has been shown to be particularly useful [20]. This methodology has its roots in prior studies involving the creation of “epigenetic clocks”, which were based on the correlation between methylation patterns and age [21,22,23]. From these studies, several authors have tried to accurately determine age from biological fluids (blood, saliva, semen) [24,25,26,27,28]. However, these studies were developed from the point of view of criminalistics: determination of the age of perpetrator from body fluids at the crime scene. There have been a few studies performed for forensic anthropological purposes, such as estimation of the age of skeletal remains. The first study was carried out by Bekaert et al. [25] on dentin samples, applying pyrosequencing and obtaining an accuracy of ±4.9 years. Giuliani et al. [29] analyzed the methylation status of three genes in different dental tissues (cementum, dentin, and pulp) obtaining accuracies between 1.2 and 7.1 years, by applying EpiTYPER, a MALDI-TOF mass spectrometry-based method. Recently, Márquez-Ruiz et al. [30] also applied pyrosequencing to determine the age in teeth based on three genes, achieving accuracies between 4.8 and 6.9 years. Correia-Dias et al. [31] applied two methodologies for methylation analysis—Sanger sequencing and SNaPshot—obtaining accuracies of 2.6 in bones and 11.4 years in teeth with the former, and 7.2 years in bones and 7.1 years in teeth with the latter. These last two studies [30,31] analyzed methylation on the whole tooth, not distinguishing between tissues. Recent work comparing methylation differences between types of dental tissues [29] indicate that isolating specific dental structures, such as pulp, might improve accuracy.

Teeth are the hardest structures in the human body, and they can survive after everything else has decomposed [15]. In particular, dental pulp is protected from external insults by hard tissues. Pulp is located in the central region of the tooth and is formed by a stromal tissue containing nerves, blood, and lymphatic vessels. The unique location and structure make it the preferred tissue for DNA analysis [32,33,34].

Based on this premise, and the need to expand the application of DNA methylation in forensic anthropology, the aim of this study was to analyze methylation patterns in pulp tissue from adult individuals and correlate these patterns with age, improving current age-at-death estimates.

## 2. Results

### 2.1. CpG Sites Identified and Individual Correlations with Age

Twenty healthy erupted third molars, ages between 22 and 70 were used in this study. After DNA extraction from the pulp, bisulfite conversion, and PCR amplification, a total of 46 CpGs sites located at ELOVL2, KLF14, SCGN, NPTX2, and FHL2 were identified, and their methylation levels were assessed through pyrosequencing. Correlation coefficients of these CpGs sites with age were calculated (Table 1). Positive and significant correlations were found in six ELOVL2 CpGs sites, with r values between 0.308 and 0.365. However, CpG5 produced an r = 0.240 and was not significant. In contrast to these results, among seven CpGs evaluated on KLF14, only one, CpG7, showed a positive and significant correlation with age (r = 0.468). The same thing happened with NPTX2, as only CpG4 showed a significant and positive correlation with age (r = 0.327). Correlations for the methylation levels for CpGs located in FHL2 with age were negative. However, while two loci in FHL2, CpG1 and CpG2, did show a strong correlation with age (r = −0.367 and r = −0.376, respectively), the results were not significant.

### 2.2. Construction of Prediction Models for Age Estimation

A backward stepwise multiple linear regression analysis was performed to create prediction models for age estimation based on an assessment of these five genes, by including sets of individual CpGs producing r values higher than 0.2, and selected after assessment on each gene of individual and significant CpG contribution to age. These models are shown in Table 2. The model with the highest correlation coefficient (R^2^ = 0.975) produced a Mean Absolute Error (MAE) between chronological and estimated age of 1.5474. This model included the majority of the ELOVL2, NPTX2, KLF14, SCGN CpGs, as well as certain CpGs from FHL2. Despite the fact that some individual CpGs of FHL2 produced correlations that were less discriminatory than those produced by other CpGs sites, they did appear to improve age estimation when combined with the other markers.

The second model removed KLF14 CpG7, obtaining a significant and strong correlation coefficient (R^2^ = 0.972) and an MAE between chronological and estimated age of 1.711. The next model eliminated FHL2 CpG6, obtaining a high correlation coefficient of R^2^ = 0.961, and an MAE between chronological and estimated age 2.047. The last model removed ELOVL2 CpG5, (*p* = 0.0001), producing an R^2^ = 0.955, and an MAE of 2.1313.

**Figure 1:**
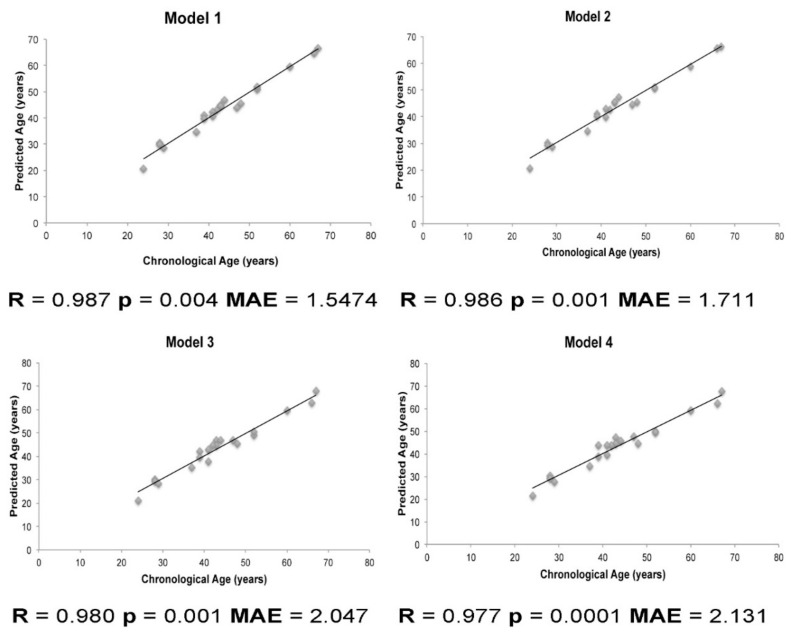
Predicted versus chronological age, determined by each statistical model. The graphs depict the accuracy of each model to predict the age in twenty pulp samples, assessed by Pearson correlations. MAE, Mean Absolute Error.

Due to the sample size (20 teeth in total), leave-one-out cross-validation was chosen to validate the models. Using this procedure, Model 1 showed the highest estimation error, followed by Model 4. Cross-validation of Models 2 and 4 produced the best accuracy. Additionally, Pearson correlations were carried out to compare the predictive ages and chronological ages, resulting in nearly the same correlation in the first three models (r = 0.98) and a slightly lower correlation in the fourth model (r = 0.97). These correlations between predictive and chronological ages are depicted in Figure 1.

## 3. Discussion

The objective of this study was to improve age-at-death estimates in forensic anthropology, based on epigenetics, particularly, the assessment of DNA methylation. This was supported by previous work applied to this discipline [25,29,30,31]. Teeth are the hardest tissues in human body, thus the present study focused on pulp, as it is the inner layer of the teeth, well protected from environmental insult. The methodological approach was based on bisulfite-modified PCR and pyrosequencing. Applying this technique, it was possible to evaluate the DNA methylation levels of five genes (ELOVL2, NPTX2, KLF14, SCGN, and FHL2). Certain CpGs of these genes showed a significant correlation with age, particularly in the case of ELOVL2. After statistical analysis, four multivariate regression models were proposed based on differential methylation of these genes. These equations were then utilized to accurately determine the age, with MAEs between 1.5 and 2.13 years.

There are few studies focused on the analysis of DNA methylation in teeth for age estimation. The first, developed by Bekaert et al. [25], used dentin samples and assessed four genes (ASPA, PDE4C, ELOVL2, and EDARADD), proposing a quadratic regression model (R^2^ = 0.74) and Mean Absolute Deviation (MAD) of 4.86 years. This estimation error was higher than the one obtained in the present study; however, the difference between tissues and the genes evaluated in both works should be considered. In fact, Giuliani et al. [29] pointed out to this difference on age estimates among the three dental layers, cementum, dentin, and pulp, assessing three genes (ELOVL2, PENK, and FHL2). As revealed in our study, Giuliani et al. demonstrated that multivariate models presented the most accurate age estimate, with the best correlation with pulp (difference between chronological and predicted age 2.25 years), and the worst correlation with dentin (difference between chronological and predicted age 7.07 years). A combination of cementum and pulp analyses retrieved the best estimation (difference between chronological and predicted age 1.20 years). Our work improves age estimates in pulp by expanding the analysis to other genes and utilizing more CpGs sites.

Unlike previous studies that have demonstrated a difference in age estimation among tooth layers, more recent work examining teeth utilized the DNA methylation levels of the whole tooth for age correlations. The study of Márquez-Ruiz et al. [30] developed multivariate models based on three genes (ELOVL2, ASPA, and EDARADD). As in the present study, certain CpGs that were less significant but still correlated with age were included in the models, improving the estimates, and providing a MAE between 4.80 and 5.08 years. Additionally, these results demonstrated that the type of tooth and sex did not have an impact on the estimates. The current work only used third molars, as they are the most protected teeth in the jaw.

Correia Dias et al. [31] also used the whole tooth to assess DNA methylation levels, using two different methodologies: Sanger Sequencing and SNaPshot. In contrast to other studies, results with EDARADD and MIR29B2C using Sanger sequencing showed no significant correlation with age, while ELOVL2, FHL2, and PDE4C showed weak correlations. However, their ELOVL2 correlations were similar to the ones obtained in the present study and previous works [25,30]. In addition, the proposed model was a simple linear regression, providing estimates with FHL2 CpG4 with a MAD of 11.35 years. When they assessed the genes by SNaPshot, FHL2 showed no significant age correlation, but again, ELOVL2 showed significant correlation with age as did KLF14, and TRIM59. These correlations were higher than those obtained in the present study. However with SNaPshot, the best model utilized a multivariate linear regression combining ELOVL2 and KLF14, producing a MAD of 7.07.

The above two works showed larger differences between chronological and estimated age, probably due to use of the whole tooth for the analysis instead of separating the layers [29]. The difference in composition among the layers and the difference in types of cells, may impact the methylation status of the chosen genes. Another important issue could be differences in methodology. To our knowledge, this study is the first work that applies pyrosequencing to assess DNA methylation in pulp tissue. The correlations obtained in this study were similar to the ones presented on Márquez-Ruiz’s work [30] as well as Bekaert et al. [25], using pyrosequencing, and to some extent the ones obtained using Sanger sequencing by Correia Dias et al. [31]. Correia Dias et al. found differences in methylation when using SNaPshot [31], producing different correlations than those obtained with Sanger sequencing. The work of Giuliani et al. [29], applying MALDI-TOF mass spectrometry, found the highest correlations with ELOVL2 and FHL2, and included more CpGs than the previous work. Thus, the technique used may have an influence on the results, particularly if bias in methylation levels occur due to poor coverage or lower input levels. However, as the present study demonstrates, ELOVL2 [25,29,30,31] and FHL2 [29,31] can produce useful age estimation in teeth.

ELOVL2 encodes a transmembrane protein involved in the synthesis of long ω3 and ω6 polyunsaturated fatty acids (PUFA) [35] and has been included on several studies for age estimation for criminalistics applications [27,36,37,38,39,40]. In fact, Zbiec-Piekarska et al. [41] assessed the usefulness of ELOVL2 and two of its CpGs sites as unique markers for age estimation in blood samples, obtaining prediction errors of approximately ±7 years. Although this determination is better than current forensic anthropological estimates, [25,29,30], more accurate determinations may be obtained by including additional genes as shown in this and other studies.

In the present study, additional genes were selected based on the composition of pulp, which contains blood capillaries, nerves, and odontoblasts [29]. FHL2, is a transcriptional co-factor involved in different processes: cell cycle regulation, bone formation, and wound healing [42,43]. Apart from the aforementioned teeth studies [29,31], its role in age estimation has been demonstrated in blood samples [31,39,40], saliva and buccal cells [27]. This work has also demonstrated the usefulness of KLF14, which was assessed in the present study, although only included in the first model. KLF14 is a member of the Krüppel-like factor family of transcription factors, regulating gene expression in adipose tissues [44]. Its usefulness in age estimation has been demonstrated in blood samples [40] and saliva [26]. The study of Alghanim et al. [45] tested this marker along with the other genes assessed in the present work, SCGN, and DLX5. In saliva, a multivariate model of two CpG sites of KLF14 retrieved a MAD of 5.8 in the training set, and the combined model of KLF14 and SCGN produced a MAD of 6.2. In contrast, the best model for blood samples was a multivariate combination of 2 CpG sites of KLF14 and 1 CpG site of SCGN, producing a MAD of 6.6. SCGN encodes for a secretagogin protein, which is involved in potassium chloride stimulated calcium flux as well as cell proliferation [46]. In the present study, SCGN seemed to play a key role in age estimation, as both CpGs 3 and 8 were included in the four models and produced among the highest correlations in the genes analyzed (except ELOVL2 CpG1 and CpG7). NPTX2 (neuronal pentraxin II) is involved in synapse formation [47], as pulp also contains nerve cells, the methylation levels of this gene may also show a response with age. Only one CpG, CpG4 was kept through the four models. Silva et al. [48] assessed the role of DNA methylation at this gene for age estimation in blood and saliva samples. Applying a multivariate regression model with 6 CpGs sites, including CpG4, they obtained an average difference of 9 years in saliva samples, with similar results for blood samples. In our results, NPTX2 played a small role (as only one CpG was selected in our models) in this determination.

The present study validated the usefulness of ELOVL2 and FHL2 as targets for age estimation in dental tissues, and also, identified new potential markers: KLF14, SCGN, and NPTX2. Compared with other methodologies for age estimation, DNA methylation provides similar results to aspartic acid racemization, as our study was able to determine the age in a range 1.5–2.13 years, depending on the model. One of the disadvantages of aspartic acid racemization is that it is influenced by temperature, so it cannot be applied to burnt remains [49]. It would be interesting to focus future studies on analyzing the effects of fire and other taphonomic effects on DNA methylation in age estimation.

The main limitation of this study is the sample size, in this case twenty teeth. This sample size could influence the significance of individual CpGs correlations with respect to age. While our sample size was similar to that used by Giuliani et al. [29], larger sample sizes can cover a finer degree of ages, possibly leading to improved models. An important point to be made is that methodology can also impact the results as discussed above. We believe that Pyrosequencing has inherent advantages over other procedures, including high coverage and precision [50], making it a useful tool to assess DNA methylation. Its relatively low cost should make it accessible to forensic labs.

## 4. Material and Methods

### 4.1. Sample Collection and Teeth Processing

Twenty healthy erupted third molars were collected from patients in a dental clinic in Spain (age range: 22–70 years old). All dental elements originated from different individuals. Florida International University’s ethical committee approved all procedures related to the experimentation with human subjects. Sample data were limited to sex, age, and population group. Teeth were washed with a soft toothbrush under running sterile distilled water and dried at room temperature. Teeth were irradiated for 15 min per side with ultraviolet light (254 nm) to eliminate exogenous DNA. Enamel and cementum were removed using a diamond brush. In the midline between cementum and enamel, crowns were separated from the roots using a diamond-cutting disc. The roots were cut along the midline and the pulp was removed using a spoon excavator.

### 4.2. DNA Extraction

DNA was extracted from pulp using DNeasy Blood and Tissue Kit (Qiagen, GmbH, Hilden, Germany), according to the manufacturer’s protocol. DNA was eluted in 35 μL Buffer AE.

### 4.3. DNA Quantification

DNA was quantified using Qubit dsDNA HS Assay Kit (Thermo Fisher Scientific, Waltham, MA, USA), according to the manufacturer’s protocol.

### 4.4. Bisulfite Conversion and PCR

200 ng of DNA was bisulfite converted using the Epi Tect Fast Bisulfite Conversion Kit (Qiagen, GmbH, Hilden, Germany). Converted DNA was eluted with 20 μL of elution buffer. 1.5 μL of converted DNA was amplified by singleplex PCR in a total volume of 0.2 μM of primers for KLF14, NPTX2, ELOVL2, FHL2, SCGN and 2x Qiagen PyroMark PCR Master Mix (Qiagen, GmbH, Hilden, Germany). All primer sequences are listed in Table S1. PCR reactions consisted of an initial hold at 95 °C for 15 min followed by 45 cycles of 30 s at 94 °C, 30 s at 56 °C, 30 s at 72 °C. PCR amplification ended with a final extension step at 72 °C for 10 min.

### 4.5. Pyrosequencing

Methylation levels were assessed after loading 15 μL of PCR product into the PyroMark Q48 Instrument (Qiagen, GmbH, Hilden, Germany), and performed pyrosequencing with 0.4 μM of sequencing primers following manufacturer’s instructions.

### 4.6. Methylation Results Analyses and Statistics

Pyrosequencing results were analyzed using the PyroMark Q48 Autoprep software (Qiagen, GmbH, Hilden, Germany). Statistical analyses were performed using IBM SPSS 26 (IBM, Armonk, NY, USA). Firstly, simple correlation analyses were performed between age and methylation levels of KLF14, NPTX2, ELOVL2, FHL2, SCGN CpGs. Multivariate linear regression models were performed to predict age. Validation of the models was performed by leave-one-out cross validation (LOOCV), in which one observation is left out and used for validation, while the remaining samples are used as a training set. This was repeated 20 times so that a complete LOOCV was performed.

## 5. Conclusions

In conclusion, the present study describes for the first time the application of DNA methylation for age determination in pulp tissues by applying pyrosequencing. The results provide great accuracy with a Mean Absolute Error (MAE) between predicted and chronological age of 1.5–2.13 years for age-at-death estimation in adult individuals. Although the mathematical models are complex, the accuracies are excellent, surpassing other techniques for forensic anthropology estimates. Apart from ELOVL2 and FHL2, we also identified three additional genes—NPTX2, KLF14, and SCGN—as potential markers for age estimation. Future research will expand these results by increasing the number of teeth, exploring other markers, and investigating taphonomic and environmental insults.

## Figures and Tables

**Table 1:** Correlation coefficients and significance of CpG sites identified in ELOVL2, KLF14, SCGN, NPTX2, and FHL2.

Gene	Site	r	*p*-Value
ELOVL2	CpG1	0.353	0.024
	CpG2	0.308	0.043
	CpG3	0.341	0.028
	CpG4	0.318	0.038
	CpG5	0.240	0.093
	CpG6	0.342	0.028
	CpG7	0.365	0.020
KLF14	CpG1	0.168	0.480
	CpG2	0.316	0.174
	CpG3	0.154	0.518
	CpG4	0.278	0.236
	CpG5	0.267	0.256
	CpG6	0.220	0.350
	CpG7	0.468	0.037
SCGN	CpG1	0.313	0.180
	CpG2	0.344	0.138
	CpG3	0.529	0.017
	CpG4	0.340	0.142
	CpG5	−0.103	0.665
	CpG6	0.268	0.254
	CpG7	0.258	0.272
	CpG8	0.508	0.022
	CpG9	0.156	0.512
	CpG10	0.291	0.213
NPTX2	CpG1	0.280	0.076
	CpG2	−0.105	0.514
	CpG3	−0.084	0.601
	CpG4	0.327	0.037
	CpG5	0.214	0.179
	CpG6	0.022	0.890
	CpG7	0.121	0.449
	CpG8	0.151	0.346
	CpG9	0.076	0.637
	CpG10	0.136	0.396
	CpG11	0.172	0.282
	CpG12	0.127	0.428
	CpG13	0.166	0.300
	CpG14	0.136	0.556
FHL2	CpG1	−0.367	0.111
	CpG2	−0.376	0.094
	CpG3	−0.251	0.285
	CpG4	−0.288	0.217
	CpG5	−0.262	0.264
	CpG6	−0.241	0.305
	CpG7	−0.088	0.713
	CpG8	−0.086	0.720

**Table 2:** Prediction models for age estimation in pulp. MAE, Mean Absolute Error; LOOCV, leave-one-out cross-validation.

Model	R	R^2^	SE	*p*-Value	MAE	MAE (LOOCV)
Age (years) = 12.763 + 4.034(ELOVL2CpG3) + 3.535(ELOVL2CpG4) − 3.040(ELOVL2CpG7) + 7.815(NPTX2CpG4) + 3.791(SCGNCpG3) + 9.122(SCGNCpG8) − 5.013(ELOVL2CpG2) − 1.643(ELOVL2CpG5) − 4.341(FHL2CpG1) + 3.571(FHL2CpG3) − 1.093(FHL2CpG4) + 3.882(FHL2CpG5) − 1.229(FHL2CpG6) − 1.662(KLF14CpG7)	0.987	0.975	3.671	0.004	1.5474	2.128
Age (years) = 14.710 + 3.675(ELOVL2CpG3) + 3.972(ELOVL2CpG4) − 2.978(ELOVL2CpG7) + 5.278(NPTX2CpG4) + 4.044(SCGNCpG3) + 8.378(SCGNCpG8) − 4.853(ELOVL2CpG2) − 1.875(ELOVL2CpG5) − 4.273(FHL2CpG1) + 3.547(FHL2CpG3) − 1.145(FHL2CpG4) + 3.640(FHL2CpG5) − 0.937(FHL2CpG6)	0.986	0.972	3.505	0.001	1.711	1.706
Age (years) = 14.349 + 4.635(ELOVL2CpG3) + 3.049(ELOVL2CpG4) − 3.681(ELOVL2CpG7) + 5.254(NPTX2CpG4) + 3.810(SCGNCpG3) + 9.503(SCGNCpG8) − 4.835(ELOVL2CpG2) − 0.982(ELOVL2CpG5) − 4.191(FHL2CpG1) + 3.778(FHL2CpG3) − 1.447(FHL2CpG4) + 2.638(FHL2CpG5)	0.980	0.961	3.874	0.001	2.047	2.083
Age (years) = 14.854 + 5.139(ELOVL2CpG3) + 2.249(ELOVL2CpG4) − 4.086(ELOVL2CpG7) + 6.927(NPTX2CpG4) + 3.505(SCGNCpG3) + 10.363(SCGNCpG8) − 4.983(ELOVL2CpG2) − 4.223(FHL2CpG1) + 4.075(FHL2CpG3) − 1.562(FHL2CpG4) + 2.506(FHL2CpG5)	0.977	0.955	3.866	0.0001	2.1313	1.942

## Data Availability

The data presented in this study are available on request from the corresponding author. The data are not publicly available due to privacy issues.

## Supplementary Materials

The following are available online at https://www.mdpi.com/article/10.3390/ijms22073717/s1, Table S1: Primer Sequences.

## References

[1] Cunha E., Baccino E., Martrille L., Ramsthaler F., Prieto J., Schuliar Y., Lynnerup N., Cattaneo C. (2009). The problem of aging human remains and living individuals: A review. Forensic Sci. Int..

[2] Ubelaker D.H. (1987). Estimating age at death from immature human skeletons: An overview. J. Forensic Sci..

[3] Albert A.M., Maples W.R. (1995). Stages of epiphyseal union for thoracic and lumbar vertebral centra as a method of age determination for teenage and young adult skeletons. J. Forensic Sci..

[4] Lynnerup N., Belard E., Buch-Olsen K., Sejrsen B., Damgaard-Pedersen K. (2008). Intra- and interobserver error of the Greulich-Pyle method as used on a Danish forensic sample. Forensic Sci. Int..

[5] Pfau R.O., Sciulli P.W. (1994). A method for establishing the age of subadults. J. Forensic Sci..

[6] Demirjian A. (1973). Tooth eruption in the French Canadian child. J. Dent. Que.

[7] Gustafson G., Koch G. (1974). Age estimation up to 16 years of age based on dental development. Odontol. Rev..

[8] Lamendin H., Baccino E., Humbert J.F., Tavernier J.C., Nossintchouk R.M., Zerilli A. (1992). A simple technique for age estimation in adult corpses: The two criteria dental method. J. Forensic Sci..

[9] Brooks S.T. (1955). Skeletal age at death: The reliability of cranial and pubic age indicators. Am. J. Phys. Anthropol..

[10] Lovejoy C.O., Meindl R.S., Pryzbeck T.R., Mensforth R.P. (1985). Chronological metamorphosis of the auricular surface of the ilium: A new method for the determination of adult skeletal age at death. Am. J. Phys. Anthropol..

[11] Iscan M.Y., Loth S.R., Wright R.K. (1984). Age estimation from the rib by phase analysis: White males. J. Forensic Sci..

[12] Meissner C., Ritz-Timme S. (2010). Molecular pathology and age estimation. Forensic Sci. Int..

[13] Ohtani S., Utsunomiya J., Minoshima T., Yamamoto K. (1994). Tooth-based age estimation of an adipocerated cadaver using the amino acid racemization method. Nihon Hoigaku Zasshi Jpn. J. Leg. Med..

[14] Ohtani S., Yamamoto T. (2005). Strategy for the estimation of chronological age using the aspartic acid racemization method with special reference to coefficient of correlation between D/L ratios and ages. J. Forensic Sci..

[15] Dobberstein R.C., Huppertz J., von Wurmb-Schwark N., Ritz-Timme S. (2008). Degradation of biomolecules in artificially and naturally aged teeth: Implications for age estimation based on aspartic acid racemization and DNA analysis. Forensic Sci. Int..

[16] Sirin N., Matzenauer C., Reckert A., Ritz-Timme S. (2018). Age estimation based on aspartic acid racemization in dentine: What about caries-affected teeth?. Int. J. Leg. Med..

[17] Egger G., Liang G., Aparicio A., Jones P.A. (2004). Epigenetics in human disease and prospects for epigenetic therapy. Nature.

[18] Turan N., Katari S., Coutifaris C., Sapienza C. (2010). Explaining inter-individual variability in phenotype: Is epigenetics up to the challenge?. Epigenetics.

[19] Bocklandt S., Lin W., Sehl M.E., Sanchez F.J., Sinsheimer J.S., Horvath S., Vilain E. (2011). Epigenetic predictor of age. PLoS ONE.

[20] Declerck K., Vanden Berghe W. (2018). Back to the future: Epigenetic clock plasticity towards healthy aging. Mech. Ageing Dev..

[21] Jones M.J., Goodman S.J., Kobor M.S. (2015). DNA methylation and healthy human aging. Aging Cell.

[22] Horvath S., Raj K. (2018). DNA methylation-based biomarkers and the epigenetic clock theory of ageing. Nat. Rev. Genet..

[23] Horvath S. (2013). DNA methylation age of human tissues and cell types. Genome. Biol..

[24] Silva D., Antunes J., Balamurugan K., Duncan G., Alho C.S., McCord B. (2016). Developmental validation studies of epigenetic DNA methylation markers for the detection of blood, semen and saliva samples. Forensic Sci. Int. Genet..

[25] Bekaert B., Kamalandua A., Zapico S.C., Van de Voorde W., Decorte R. (2015). Improved age determination of blood and teeth samples using a selected set of DNA methylation markers. Epigenetics.

[26] Hong S.R., Jung S.E., Lee E.H., Shin K.J., Yang W.I., Lee H.Y. (2017). DNA methylation-based age prediction from saliva: High age predictability by combination of 7 CpG markers. Forensic Sci. Int. Genet..

[27] Jung S.E., Lim S.M., Hong S.R., Lee E.H., Shin K.J., Lee H.Y. (2019). DNA methylation of the ELOVL2, FHL2, KLF14, C1orf132/MIR29B2C, and TRIM59 genes for age prediction from blood, saliva, and buccal swab samples. Forensic Sci. Int. Genet..

[28] Correia Dias H., Cordeiro C., Corte Real F., Cunha E., Manco L. (2020). Age Estimation Based on DNA Methylation Using Blood Samples From Deceased Individuals. J. Forensic Sci..

[29] Giuliani C., Cilli E., Bacalini M.G., Pirazzini C., Sazzini M., Gruppioni G., Franceschi C., Garagnani P., Luiselli D. (2016). Inferring chronological age from DNA methylation patterns of human teeth. Am. J. Phys. Anthropol..

[30] Marquez-Ruiz A.B., Gonzalez-Herrera L., Luna J.D., Valenzuela A. (2020). DNA methylation levels and telomere length in human teeth: Usefulness for age estimation. Int. J. Leg. Med..

[31] Correia Dias H., Corte-Real F., Cunha E., Manco L. (2020). DNA methylation age estimation from human bone and teeth. Aust. J. Forensic Sci..

[32] Sweet D.J., Sweet C.H. (1995). DNA analysis of dental pulp to link incinerated remains of homicide victim to crime scene. J. Forensic Sci..

[33] Veeraraghavan G., Lingappa A., Shankara S.P., Mamatha G.P., Sebastian B.T., Mujib A. (2010). Determination of sex from tooth pulp tissue. Libyan J. Med..

[34] Potsch L., Meyer U., Rothschild S., Schneider P.M., Rittner C. (1992). Application of DNA techniques for identification using human dental pulp as a source of DNA. Int. J. Leg. Med..

[35] Leonard A.E., Kelder B., Bobik E.G., Chuang L.T., Lewis C.J., Kopchick J.J., Mukerji P., Huang Y.S. (2002). Identification and expression of mammalian long-chain PUFA elongation enzymes. Lipids.

[36] Freire-Aradas A., Phillips C., Mosquera-Miguel A., Giron-Santamaria L., Gomez-Tato A., de Cal M.C., Alvarez-Dios J., Ansede-Bermejo J., Torres-Espanol M., Schneider P.M. (2016). Development of a methylation marker set for forensic age estimation using analysis of public methylation data and the Agena Bioscience EpiTYPER system. Forensic Sci. Int. Genet..

[37] Naue J., Hoefsloot H.C.J., Mook O.R.F., Rijlaarsdam-Hoekstra L., van der Zwalm M.C.H., Henneman P., Kloosterman A.D., Verschure P.J. (2017). Chronological age prediction based on DNA methylation: Massive parallel sequencing and random forest regression. Forensic Sci. Int. Genet..

[38] Naue J., Sanger T., Hoefsloot H.C.J., Lutz-Bonengel S., Kloosterman A.D., Verschure P.J. (2018). Proof of concept study of age-dependent DNA methylation markers across different tissues by massive parallel sequencing. Forensic Sci. Int. Genet..

[39] Dias H.C., Cordeiro C., Pereira J., Pinto C., Real F.C., Cunha E., Manco L. (2020). DNA methylation age estimation in blood samples of living and deceased individuals using a multiplex SNaPshot assay. Forensic Sci. Int..

[40] Zbiec-Piekarska R., Spolnicka M., Kupiec T., Parys-Proszek A., Makowska Z., Paleczka A., Kucharczyk K., Ploski R., Branicki W. (2015). Development of a forensically useful age prediction method based on DNA methylation analysis. Forensic Sci. Int. Genet..

[41] Zbiec-Piekarska R., Spolnicka M., Kupiec T., Makowska Z., Spas A., Parys-Proszek A., Kucharczyk K., Ploski R., Branicki W. (2015). Examination of DNA methylation status of the ELOVL2 marker may be useful for human age prediction in forensic science. Forensic Sci. Int. Genet..

[42] Canault M., Tellier E., Bonardo B., Mas E., Aumailley M., Juhan-Vague I., Nalbone G., Peiretti F. (2006). FHL2 interacts with both ADAM-17 and the cytoskeleton and regulates ADAM-17 localization and activity. J. Cell. Physiol..

[43] Friedrich F.W., Reischmann S., Schwalm A., Unger A., Ramanujam D., Munch J., Muller O.J., Hengstenberg C., Galve E., Charron P. (2014). FHL2 expression and variants in hypertrophic cardiomyopathy. Basic Res. Cardiol..

[44] Small K.S., Hedman A.K., Grundberg E., Nica A.C., Thorleifsson G., Kong A., Thorsteindottir U., Shin S.Y., Richards H.B., Consortium G. (2011). Identification of an imprinted master trans regulator at the KLF14 locus related to multiple metabolic phenotypes. Nat. Genet..

[45] Alghanim H., Antunes J., Silva D., Alho C.S., Balamurugan K., McCord B. (2017). Detection and evaluation of DNA methylation markers found at SCGN and KLF14 loci to estimate human age. Forensic Sci. Int. Genet..

[46] Skovhus K.V., Bergholdt R., Erichsen C., Sparre T., Nerup J., Karlsen A.E., Pociot F. (2006). Identification and characterization of secretagogin promoter activity. Scand. J. Immunol..

[47] Moran L.B., Hickey L., Michael G.J., Derkacs M., Christian L.M., Kalaitzakis M.E., Pearce R.K., Graeber M.B. (2008). Neuronal pentraxin II is highly upregulated in Parkinson’s disease and a novel component of Lewy bodies. Acta Neuropathol..

[48] Soares Bispo Santos Silva D., Antunes J., Balamurugan K., Duncan G., Sampaio Alho C., McCord B. (2015). Evaluation of DNA methylation markers and their potential to predict human aging. Electrophoresis.

[49] Zapico S.C., Ubelaker D.H., Adserias-Garriga J. (2013). Applications of physiological bases of ageing to forensic sciences. Estimation of age-at-death. Ageing Res. Rev..

[50] Slatko B.E., Gardner A.F., Ausubel F.M. (2018). Overview of Next-Generation Sequencing Technologies. Curr. Protoc. Mol. Biol..

