# Supplementary material for: Identifying Methylation Patterns in Dental Pulp Aging: Application to Age-at-Death Estimation in Forensic Anthropology
Source: Int J Mol Sci. 2021 Apr 2;22(7):3717. doi: 10.3390/ijms22073717 (PMC8038189; doi:10.3390/ijms22073717)
Supplement: Supplementary file 1 [file ijms-22-03717-s001.pdf]

Table S1. Primer sequences to evaluate CpG sites in five different genes. *biotinylated primer. Y-location of CpGs (pyrimidine C or T); R-location of CpGs (purine A or G).

Gene	Primer	Sequence	CpG sites analyzed (in bold)
ELOVL2	Forward*	AGGGGAGTAGGGTAAGTGAGG	CC**R**TAAAC**R**TTAAACC**R**CC**R**C**R**C**R**AAACC**R**AC
	Reverse	AACAAAACCATTTCCCCCTAATAT	
	Sequencing	ACAACCAATAAATATTCCTAAAACT	
NPTX2	Forward	GGTTGTGAGAAGGTAGGAGATTT	T**Y**G**Y**GGTGTA**Y**G**Y**GATTTT**Y**GAGA**Y**GATAG**Y**G**Y**GGTTATTGTTAGTAG**Y**GAAGG**Y**GTTTTT**Y**G**Y**GGAG**Y**GTTT**Y**GA
	Reverse*	ACCAACAACCCCAACATCCC	
	Sequencing	AGAAGGTAGGAGATTTTTGTT	
FHL2	Forward	TGTTTTTAGGGTTTTGGGAGTATAG	AGTTAT**Y**GGGAG**Y**GT**Y**GTTTT**Y**GG**Y**GTGGGTTTT**Y**GGG**Y**GAGTTT**Y**GG
	Reverse*	ACACCTCCTAAAACTTCTCCAATCTCC	
	Sequencing	GGTTTTGGGAGTATAGT	
KLF14	Forward	TTTGGTGTAGTTAGGGAAGGGGTATT	TGG**Y**GTTTGGTAGTAGGTGTGATAGATTTTTTT**Y**GGGG**Y**GTTTGATT**Y**G**Y**GG**Y**GGGGG**Y**GGGGTTTGTTTTTAGGGTTTTTTAG
	Reverse*	CACCAACAACCTCTAATAAATTCTCTA	
	Sequencing	GGGAAGGGGTATTGG	
SCGN	Forward	AAGGAGTTTTTTTTAAAGTTGTTTAGG	TT**Y**G**Y**GT**Y**GGTGTTTGGTTTT**Y**GT**Y**GTTAATATTATGGATAGTTTT**Y**GGGAAT**Y**GATTTTGGGG**Y**GTTTGGA**Y**GT**Y**GTTGGTTTTTGGTAG
	Reverse*	ACAACCCAAATCCATAACTTTTCCTACA	
	Sequencing	TTTTTTTAAAGTTGTTTAGGTTTT	
